# In-Depth Analysis of the Prognostic Factors Associated with Short-Term Outcome in Equine Colic Patients: Multicentric Retrospective Study

**DOI:** 10.3390/ani16030496

**Published:** 2026-02-05

**Authors:** Irene Nocera, Dania Cingottini, Chiara Di Franco, Giulia Sala, Francesca Bindi, Alessandro Spadari, Riccardo Rinnovati, Valentina Vitale, Eduard Jose-Cunilleras, Micaela Sgorbini

**Affiliations:** 1 Department of Veterinary Science, University of Pisa, Viale delle Piagge 2, 56122 Pisa, Italy; irene.nocera@vet.unipi.it (I.N.); chiara.difranco@unipi.it (C.D.F.); giulia.sala@unipi.it (G.S.); francesca.bindi@phd.unipi.it (F.B.); micaela.sgorbini@unipi.it (M.S.); 2 Department of Veterinary Medical Sciences, University of Bologna, 40064 Bologna, Italy; alessandro.spadari@unibo.it (A.S.); riccardo.rinnovati@unibo.it (R.R.); 3 Department Animal Medicine and Surgery, Universidad CEU-Cardenal Herrera, CEU Universities, 46115 Alfara del Patriarca, Spain; valentina.vitale@uchceu.es; 4 Department of Animal Medicine and Surgery, Facultat de Veterinària, Travessera dels Turons s/n, 08193 Bellaterra, Spain

**Keywords:** obstructive non-strangulating colic, strangulating colic, predisposing factor

## Abstract

Several studies investigated risk and prognostic parameters for horses with colic; however, the consensus is still debated. The present work aimed to investigate colic outcomes and to identify risk factors in horses referred for colic. In this multicenter retrospective study, 236 clinical records of equids referred for colic at three different equine centers were reviewed. The following data were collected: history, signalment, physical examination at the time of admission, hematological and biochemical analysis, diagnosis, SIRS status and 0–6 point-scale SIRS score, colic type, treatment attempted, and outcome. Descriptive statistics were performed, and distribution of continuous variables was reported as median and percentile. A multivariable logistic regression model was applied to assess parameters associated with colic outcomes in horses (*p* < 0.05). A total of 138/236 horses were included in the study. The univariate analysis identified as potentially associated with the outcome: sex (*p* = 0.046), colic type (*p* < 0.001), treatment type (*p* < 0.001), SIRS score (*p* = 0.049), age (*p*-value = 0.057), heart rate (*p* = 0.013), and respiratory rate (*p* = 0.017). The logistic regression model indicated that colic type (*p* < 0.001) and age (*p* = 0.004) were significantly associated with a negative outcome. Equine colic risks are multifactorial; prognosis declines with age and strangulating obstructive non-strangulating colic. Poor outcomes link to cardiovascular signs like elevated heart rate, SIRS status and score, and blood lactate. Heterogeneity from diverse sites limits generalizability, but standardized protocols, binarized data, and a multicenter approach enhance robustness and representativeness while reducing local biases.

## Simple Summary

Numerous studies have investigated risk factors for equine colic and prognostic indicators, yet consensus remains elusive due to methodological variations. This retrospective analysis reviewed medical records from 236 colic cases across three equine clinics, collecting data on signalment (age, breed, sex), history, clinical exams, hematology, SIRS status and 0–6 point-scale SIRS score, lesion types (obstruction vs. strangulating), treatments (medical vs. surgical), and survival outcomes. Multivariate regression confirmed strangulating lesions and older age as primary predictors of mortality, reflecting ischemia, necrosis, and comorbidities. Multifactorial etiology of colic complicates management. Geriatric horses and vascular compromises heighten risks, amplified by cardiovascular instability, SIRS escalation (26–60% fatality), and lactate surges signaling hypoperfusion. These findings underscore serial monitoring for triage, though retrospective biases necessitate prospective validation.

## 1. Introduction

Colic refers to abdominal pain and is the main cause of emergency veterinary care in horses. While colic cases are mostly due to acute gastrointestinal disease, other abdominal issues like urinary or genital tract disorders can also cause colic [1].

Colic represents one of the primary reasons for morbidity and mortality in equine populations, striking between 3.5% and 11% of horses annually with significant health implications. Roughly 11% of horses that develop colic ultimately do not survive the condition, highlighting its severity. The predominant cause of death in these cases stems from extensive ischemic injury to the intestines, which frequently arises as a complication of strangulating obstructions impacting about 21% of colic-affected horses referred to specialized veterinary hospitals [2].

Studies show that inflammatory or strangulating intestinal diseases can lead to systemic inflammatory response syndrome (SIRS) [3,4]. A severe SIRS scoring model including historic SIRS criteria plus lactate and mucous membrane status proposed by others reported elevated mortality risk in horses with scores higher than 3 [5]. Early diagnosis and treatment of SIRS are vital to improve outcomes, but common SIRS criteria in adult horses’ lack specificity and lead to misclassification [4].

In horses with colic, breakthrough biomarkers for diagnosing and predicting colic include systemic/peritoneal L-lactate (suggestive of strangulating intestinal lesions and ischemia), alongside elevated SAA links to colic/inflammation (degree varies by trigger), aiding prognosis, surgery needs, infections, and lesion distinction. Innovative biomarkers like procalcitonin (PCT) (higher in colic vs. healthy; sensitive for intestinal ischemia) and protein carbonylated content (PCC) (cutoff 0.049 nmoL/mL/mg, sens/spec 74.5%/72.2% for SIRS) promise rapid/accurate diagnostics/prognostics [6,7,8,9,10,11,12]. In particular, horses with strangulating intestinal lesions showed a markedly elevated mean peritoneal lactate concentration (8.45 ± 5.52 mmol/L) compared to those with non-strangulating lesions (2.09 ± 2.09 mmol/L) [6]. In equine medicine, veterinarians currently rely mainly on blood and peritoneal lactate to help identify intestinal ischemia in horses suffering from colic. While this marker effectively distinguishes between surgical and non-surgical intestinal disorders, they lack perfect predictive accuracy. Therefore, ongoing research into additional biomarkers for detecting intestinal injury and ischemia remains essential [2,7].

Colic in horses might have different etiology, and most of the cases resolve with medical treatment. However, 8–20% need hospitalization, intensive care, and often a definitive diagnosis frequently necessitates an exploratory laparotomy. Thus, equine clinicians are inherently constrained in the extent of information they can provide to horse owners concerning prognosis and potential outcomes without establishing a confirmed diagnosis. Risk factors include age, breed, sex, diet changes, exercise, housing, and parasites [13,14,15]. Treatment decisions and prognosis consider pain response, gastric reflux, cardiovascular status, peritoneal fluid analysis, and rectal exams, alongside owner factors and facility resources [13,14,15].

Recognizing factors that heighten the risk of abdominal pain in horses proves crucial for owners and veterinarians alike, as such insights enable early identification of vulnerable animals and guide preventive management practices. Evidence on these risk factors supports targeted strategies to minimize or avert the condition entirely. Numerous studies have explored these elements, yielding a broad array of publications that employ diverse methodologies [1]. Several studies have investigated risk factors and prognostic parameters for the assessment of horses with colic; however, to date, the consensus on standardized and universally accepted parameters is still debated [16,17,18].

The present work aimed to investigate the parameters associated with colic outcomes and to identify risk factors in horses referred for colic syndrome to different veterinary hospitals providing secondary health care. We hypothesized that clinical parameters could be associated with colic severity and outcome of horses admitted to veterinary clinics.

## 2. Materials and Methods

### 2.1. Cases

In this multicenter retrospective study, data were reviewed from clinical records of equids referred for colic syndrome at three different equine referral centers, which were all veterinary teaching hospitals providing secondary horse health care (Veterinary Teaching Hospital (VTH), University of Pisa, Italy; VTH, University of Bologna, Italy; VTH, Universitat Autònoma de Barcelona, Spain. Medical records registered during a three-year period (2017–2019) were reviewed. The owner’s written consent was obtained for all the horses included in this study.

The following data were mandatory to include clinical records in the study: (1) signalment (species, breed, age, and sex); (2) physical examination findings at admission: heart rate, respiratory rate, temperature, mucous membrane color and capillary refill time, hydration status, abdominal auscultation, nasogastric intubation and abdominal palpation per rectum results, pain assessment via behavioral scores; (3) laboratory data: hematological parameters (packed cell volume and total white blood cell count), blood lactate; (4) diagnostic information detailed imaging (ultrasound, radiography); (5) colic type (obstructive non-strangulating and strangulating) [6]; (6) treatment attempted (medical/surgical); and (7) outcome. Starting from the included data, the SIRS status (Table 1) [5] was assessed as follows: presence of abnormal leukocyte count or distribution as leukopenia, leukocytosis (lower than 5 or higher than 12.5 × 10^3^/μL) or higher than 10% band neutrophils, hyperthermia or hypothermia (lower than 37 °C or higher than 38.5 °C), tachycardia (higher than 52 bpm), and tachypnea (higher than 20 bpm). Horses with 0 or 1 abnormal criterion were considered SIRS-negative, while those with 2 or more abnormal criteria were considered SIRS-positive. Moreover, the 0–6 point-scale SIRS score (Table 1) was also calculated [5], using the blood lactate concentration (normal blood lactate: lower or equal to 2.06 mmol/L vs. abnormal higher than 2.06 mmol/L) and color of the mucous membranes (normal mucous membranes: pink and moist vs. abnormal mucous membranes: injected, purple, muddy, toxic, red, or white). Blood lactate concentration was assessed from whole blood, immediately analyzed after withdrawal with Accutrend Lactate, Roche Diagnostics S.p.S. (Monza, Italy).

Cases with inflammatory gastrointestinal or urogenital tract diseases were not included. The time elapsed from the onset of symptoms to the time of admission or the pre-admission treatments were not recorded.

### 2.2. Statistical Analysis

The statistical analysis was conducted using IBM SPSS v. 29.0 (IBM Corporation, Armonk, NY, USA). Descriptive statistics are shown after classifying cases as surviving and non-surviving horses. The Shapiro–Wilk test was used to assess the distribution of continuous variables. Since these variables were not normally distributed, they were reported as median, and 25–75th percentile (25–75°P). Categorical variables were presented as frequencies and percentages.

The outcome was binarized as discharged (survival to hospital release) versus non-discharged (euthanasia or death), enabling prognostic modeling. To investigate the parameters associated with colic outcomes in horses, a multivariable logistic regression model was applied. First, a univariable analysis was conducted to identify potential factors associated with the outcome, considering a significance threshold of *p* < 0.1. Variables identified as potential risk factors were then included in a multivariable logistic regression model, where backward stepwise elimination was used to determine the significant parameters (*p* < 0.05) associated with the outcome. To account for the multicenter design, center was explored as a potential source of heterogeneity. First, the distribution of outcomes across centers was assessed using a chi-square test. Second, center was evaluated within multivariable logistic regression models by testing interactions between center and the main predictors. Given the limited number of outcome events, center was not retained as an independent fixed effect in the final model unless statistically or clinically relevant. Missing data were assessed for all variables. Variables with more than 10% missing values were excluded from the multivariable analysis. Given the low proportion of missing data in the remaining variables, no imputation was performed, and a complete-case analysis was applied. Collinearity among candidate predictors was assessed using Spearman’s rank correlation. When pairs of variables showed a correlation coefficient greater than 0.60, only one variable was retained for further analysis based on clinical relevance. Model performance was evaluated using Nagelkerke pseudo-R^2^. Results are reported as odds ratios (OR) with corresponding 95% confidence intervals (CI).

## 3. Results

A total of 236 records were reviewed, and a total of 138/236 horses hospitalized for colic were included in this study, of which: 100/138 were collected from VTH, University of Bologna, Italy; 23/138 were collected from VTH Universitat Autònoma de Barcelona, Spain; and 15/138 were collected from VTH, University of Pisa, Italy.

The majority of the horses were crossbreeds (n = 87/138, 63%), followed by Quarter Horses (n = 14, 10.2%), Trotters (n = 13, 9.4%), Ponies (n = 8, 5.8%), Draft horses (n = 8, 5.8%), Donkeys (n = 4, 2.9%), Arabians (n = 3, 2.2%), and Shetland Ponies (n = 1, 0.7%). Most of the horses were female (n = 62/138, 44.9%), followed by geldings (n = 48/138, 34.8%) and stallions (n = 28/138, 20.3%).

Of the cohort of 138 horses, 109/138 (79%) survived, and 29/139 (21%) died. The median age among surviving horses was 12 years old (range 6–17.5 years old), and the median age of non-surviving horses was 15 years old (range 9.5–22.7 years old).

Among the surviving horses, 52/109 were submitted to surgery, while 57/109 were medically treated. Of the surviving horses submitted to surgery, 31/52 were affected by strangulating colic, while 21/52 were affected by obstructive non-strangulating colic. Of the surviving horses medically treated, 57/57 were affected by obstructive non-strangulating colic.

Among the non-surviving horses, 28/29 were humanely euthanized not for financial constraints (24 in Bologna and 4 in Barcelona), and 1/29 spontaneously died (in Bologna). No horses were euthanized or died in Pisa. Of the euthanized/dead horses, 26/29 were submitted to surgery, and 3/29 were medically treated. Of the horses submitted to surgery, 21/26 were affected by strangulating and 5/26 by obstructive non-strangulating (two right dorsal displacement, one cecal impaction, one large colon impaction) colic. Of the horses medically treated, 1/3 was affected by strangulating (small intestine torsion) and 2/3 by obstructive non-strangulating (one right dorsal displacement and one large colon impaction) colic.

Regarding the SIRS status at the time of hospitalization, 65/109 (59.6%) of the surviving horses were SIRS-negative, while 44/109 (40.4%) were SIRS-positive. Among the deceased horses, 14/29 (50%) were SIRS-negative, and 15/29 (50%) were SIRS-positive at admission. The SIRS score values of surviving and non-surviving horses are reported in Table 2. The data on continuous variables, divided between surviving and deceased animals, are reported in Table 3.

The univariate analysis identified several parameters as potentially associated with the outcome, including sex (*p* = 0.046), colic type (*p* < 0.001), treatment type (*p* < 0.001), SIRS score (*p* = 0.049), age (*p*-value = 0.057), heart rate (*p* = 0.013), and respiratory rate (*p* = 0.017). Among the variables identified as potentially associated with outcome at univariable analysis, no relevant collinearity was detected among predictors included in the multivariable model. Only blood lactate and white blood cell count showed missing values, corresponding to five cases (3.6% of the total sample). Given the low proportion of missing data in the remaining variables, no imputation was performed, and a complete-case analysis was applied.

The logistic regression model built with the above variables indicated that only colic type (*p* < 0.001) and age (*p* = 0.004) were significantly associated with a negative outcome. In particular, animals with obstructive non-strangulating colic had an odds ratio of 16.04 times higher for survival compared to horses with strangulating colic, and younger horses had an odds ratio of 0.9 for survival compared to older ones (Table 4). In exploratory models including interactions between center, no consistent center-related effect was detected for colic type and age. Given the absence of an overall center effect and the limited sample size per center, center was not included as a fixed effect in the final multivariable model. When expressed in clinically meaningful increments, a 5-year increase in age was associated with a 38% decrease in the odds of survival (OR = 0.62, CI 95% = 0.42–0.86). The final multivariable logistic regression model showed satisfactory explanatory power, with a Nagelkerke pseudo R^2^ of 0.38.

## 4. Discussion

This retrospective study systematically examined medical records from horses diagnosed with colic syndrome, aiming to pinpoint key risk factors and prognostic indicators influencing clinical outcomes. By reviewing historical data from affected animals across multiple clinics, the analysis focused on identifying patterns in patient characteristics, clinical presentations, and therapeutic responses that correlate with survival or complications. Overall, several parameters emerged as potentially associated with adverse outcomes. However, only older horses and colic type (i.e., strangulating colic) were significantly associated with a negative outcome.

Age is often considered an indicator of colic severity in horses, but its impact on prognosis is still inconsistent [19,20,21]. The studies employed varied methodologies, with most adopting different age ranges and reference categories. In accordance with the present findings, some studies showed that older horses have lower survival rates. Rising age in horses emerged as a notable risk factor across studies in a scoping review [1], in agreement with the present study where a 5-year increase in age was associated with a 38% decrease in the odds of survival (OR ≈ 0.62). However, other studies found no significant difference or even suggested that age can be protective [19,20,21]. These mixed findings may be due to older horses commonly having other health issues or conditions and variations in treatment approaches based on age. Mature horses aged 10 to 14 years usually have the best outcomes [21], likely because young and geriatric horses have different predisposing factors often complicated by concurrent diseases. Geriatric horses tend to have more severe gastrointestinal disease and overall lower short-term survival [15,19,20]. The horse’s age might be considered critical in determining outcomes [15,19,21]. Further evaluation of this evidence is required to pinpoint the most vulnerable age groups, though upcoming research must prioritize methodological uniformity to facilitate synthesis of findings [1].

In the present study, strangulating colic was associated with poor prognosis. As previously reported, strangulating colic in horses, characterized by vascular occlusion and resulting tissue ischemia, yields worse prognosis compared to non-strangulating forms, featuring short-term survival to hospital discharge of 50–80% after surgery, which declines markedly in critical scenarios such as 360° large colon volvulus (approximately 36%) [21]. The primary lesion significantly influenced the outcome in colic cases, with lesions such as large colon volvulus, small intestinal volvulus, and pedunculated lipoma associated with a higher risk of poor prognosis [21,22,23]. Strangulating lesions frequently require resection and anastomosis, which is linked to lower short-term survival rates in horses after colic surgery. Horses that do not undergo anastomosis have about a 2.3 times greater chance of survival compared to those that do [21,22,23]. Intraoperative euthanasia affects 25–39% of cases of irreparable lesions or extensive necrosis. Survival post-recovery reaches 79–83%, and long-term survival falls to 80–92% at 1–2 years, due to sequelae including endotoxemia, laminitis, or need for repeat surgery [17]. Overall, survival rates for horses with strangulating lesions are lower than for those with non-strangulating lesions, with survival approximately 68–75% short-term and reduced long-term survival depending on severity [16,24,25,26,27]. Thus, the type and location of the lesion affect the prognosis, with strangulating lesions particularly linked to worse outcomes. Moreover, in the present study horses undergoing surgical treatment showed poor outcomes compared to those who received medical management. This has been previously reported to be mainly due to elevated complication rates and the selection of more severe cases requiring intervention, despite achieving 50–80% short-term survival in numerous instances [2]. Horses subjected to laparotomy commonly encounter postoperative complications, including colic or pain (28–58%), incisional infections or drainage (26–44%), ileus (14%), endotoxemic shock (12%), peritonitis (3–31%), and thrombophlebitis (8%); small intestinal procedures, particularly resections, further increase susceptibility to adhesions, reflux, recurrent colic, and relaparotomy needs [2].

Among all parameters investigated, the univariate analysis identified several parameters as potentially associated with the outcome including: sex (with geldings showing heightened vulnerability in this cohort), heart rate (elevated levels signaling cardiovascular stress and hypoperfusion), respiratory rate (tachypnea reflecting systemic inflammation or shock), SIRS score (higher values indicating escalating inflammatory cascades and multi-organ risks), and treatment type (surgical interventions linked to greater complication burdens despite necessity in severe cases).

The role of sex in equine colic is debated due to heterogeneous study conditions and outcomes. In the present study, geldings resulted potentially associated with adverse prognosis. However, the literature reveals mixed evidence [18,28]: males, especially intact stallions and geldings, exhibit elevated risks for small intestinal entrapments and surgical colics, linked to workload, anatomy, overexertion, and hernias (e.g., 77% male cases in working horses; 55% surgical in stallions). Mares show higher post-foaling vulnerability to large colon torsion [18,28]. However, sex, weight, and breed lack prognostic significance in regression models. A recent study reported that sex effects often intertwine with breed, activity, and management, e.g., stallions in Indonesia showed higher risk, along with recent diet changes and poor body condition score [18].

In the present study, horses presenting with elevated heart rate (HR) and respiratory rate (RR) at admission exhibited adverse outcomes. Admission and post-treatment elevations in HR, packed cell volume (PCV), and prolonged capillary refill time (CRT) were reported to strongly correlate with non-survival, underscoring their prognostic utility [15,18]. PCV and HR are pivotal colic indicators, reflecting dehydration, hemoconcentration from gastrointestinal fluid sequestration, stress, pain, shock, or endotoxemia [28]. Elevated PCV and tachycardia at admission predict surgical necessity, particularly in small intestinal strangulations with protracted symptoms [18,28,29]. Surgical candidates displayed higher HR and PCV than medical cases; non-survivors had greater values and longer colic duration. The literature confirms non-survivors often exceed PCV > 50%, HR > 60 bpm, with delayed referral worsening prognosis [18,28,29]. Serial monitoring is essential: rising trends warrant intervention escalation, while PCV normalization (37–42%) signals recovery [18,28,29]. These parameters constitute reliable early prognostic markers for management decisions.

Elevated RR at admission similarly portends poor prognosis, associating with reduced survival via pain, shock, endotoxemia, or hypoperfusion [30]. Tachypnea (>30–40 breaths/min) accompanies tachycardia and hyperlactatemia, featuring in scoring systems like the Colic Assessment Score (CAS), where it diminishes odds [30].

This study links SIRS scores > 3 at admission to adverse outcomes in equine colic. The SIRS score is a validated prognostic tool, with escalating values (≥2 criteria) denoting intensified systemic inflammation, endotoxemia, intestinal ischemia, bacterial translocation, and multi-organ dysfunction, conferring 8–20-fold increased mortality odds [5]. Colic severity—e.g., compromised bowel length, abdominal chronicity, strangulations—predisposes to SIRS development and complications [31]. Mortality escalates markedly: 26% at score 2, 60% at 3–4; survival shortens from days to hours [5]. Individual parameters (e.g., HR, RR) lack specificity due to multifactorial overlap, but composite SIRS scoring amplifies predictive accuracy by integrating synergistic data, minimizing errors [5]. Many models incorporate SIRS criteria for survival prognostication [29,30,32,33]. Enhanced models combine SIRS with lactate > 4 mmol/L (hypoperfusion marker) and mucous membrane derangements (hyperemia, cyanosis, toxic lines), yielding ~90% sensitivity/specificity for non-survival or surgical needs. The CAS exemplifies this: logistic regression identified six variables—HR, RR, lactate, total calcium, abnormal ultrasound, rectal exam—for survival prediction. Prospective scores > 7 predicted death (sensitivity 84%, specificity 62%, PPV 88%, NPV 52%) [30,31,32,33,34]. SIRS-positive horses, especially surgical candidates, face < 50% survival, guiding triage: aggressive fluids, antimicrobials, and laparotomy. Serial monitoring is crucial for normalization of vitals and leukograms signals recovery; persistence forecasts fatality [5,10]. Thus, SIRS underpins robust, dynamic prognostic stratification in colic management.

The retrospective nature of this study has inherent limitations that undermine reliability and generalizability. In particular, biases as selection (non-random case inclusion), recall (inaccurate historical reporting), euthanasia decisions (owner decision, financial constraints, perceived prognosis) and observer bias from limiting the possibility of retrieving full data for all of the medical reports. Moreover, the lack of information on time of referral, which would have added precious information to critically analyze and compare outcomes and complications. The heterogeneity of cases collected from different locations and clinics represents an inherent limitation of this multicenter retrospective study. Differences among centers in terms of referral patterns, case mix, local patient populations, seasonal variations, and clinical management strategies may contribute to selection bias and environmental confounding, potentially limiting the generalizability of the results to broader contexts. However, in the present dataset, no significant overall association between center and outcome was detected, suggesting that center-specific differences did not substantially influence short-term survival. Taken together, these findings indicate that, while some degree of inter-center variability is unavoidable in multicenter studies, the primary prognostic associations identified in this analysis appear largely robust across centers. In contrast, in the present study standardization and collaboration were promoted using unified protocols to standardize diagnostic and scoring [5], collecting and recording data (i.e., outcome, survival, etc.) using binarized setting, to enhance prognostic modeling. These elements would provide high-quality data and scientific networking. Additionally, multicenter studies offer greater representativeness by recruiting from diverse clinics to capture real-world variability in patients and settings; these would help boost statistical power through larger cumulative samples and enable reliable subgroup analyses; and provide cross-validation via inter-center comparisons to ensure result consistency and minimize local biases.

Additionally, given the limited number of non-survivors, the risk of model overfitting was carefully considered. Although several variables were explored at univariable analysis, the final multivariable model was highly parsimonious, retaining only two clinically plausible predictors. This resulted in an adequate events-per-variable ratio, thereby reducing the risk of overfitting. Nevertheless, the retrospective design and the limited sample size may have affected both statistical power and generalizability. Increasing the sample size would enhance the robustness of the estimates and allow more reliable subgroup analyses. Therefore, further prospective studies including larger and well-characterized populations are warranted to improve representativeness, extrapolate findings to broader target populations, and confirm the identified prognostic associations.

## 5. Conclusions

This retrospective study on equine colic identifies key prognostic factors including sex (geldings at higher risk), age (older horses vulnerable), elevated heart and respiratory rates, high SIRS scores (>3), strangulating lesions, and surgical treatment needs, all linking to poorer outcomes like non-survival or complications. Conflicting evidence persists regarding the effect of sex due to study variations, with males showing trends toward surgical colic but intertwined with management. In addition, vital signs (HR > 60 bpm, PCV > 50%, RR > 30–40 bpm) and SIRS signal dehydration, shock, and inflammation, demanding serial monitoring for intervention guidance. Older age and strangulating types worsen prognosis via necrosis and resection risks (50–80% short-term survival). Retrospective biases (selection, data gaps) and missing referral times limit generalizability, warranting prospective validation for clinical use.

## Figures and Tables

**Table 1:** Clinical variables used to calculate systemic inflammatory response syndrome (SIRS) status and SIRS score [5].

Variable	Normal	Abnormal
Abnormal leukocyte count	5–12.5 × 10^3^/μL	<5 × 10^3^/μL or >12.5 × 10^3^/μL or >10% band neutrophils
Temperature	37–38.5 °C	<37 °C or >38.5 °C
Heart rate	≤52 bpm	>52 bpm
Respiratory rate	≤20 bpm	>20 bpm
Blood lactate	≤2.06 mmol/L	>2.06 mmol/L
Mucous membrane	pink and moist	injected, purple, muddy, toxic, red, white

**Table 2:** Frequency and percentage of SIRS score in surviving and non-surviving colic horses in a clinical setting.

SIRS Score	Survivor (n = 109)	Non-Survivor (n = 29)
0	25/109 (22.9%)	2/29 (7%)
1	21/109 (19.4%)	3/29 (10.3%)
2	20/109 (18.3%)	5/29 (17.2%)
3	20/109 (18.3%)	9/29 (31.0%)
4	19/109 (17.4%)	6/29 (20.7%)
5	4/109 (3.7%)	3/29 (10.3%)
6	-	1/29 (3.5%)

**Table 3:** Median (25th and 75th percentile) of continuous variables recorded at admission to the hospital of 138 colic horses.

	Survived (n = 109)	Not-Survived (n = 29)
Age (years)	12 (6–18)	12.5 (7.7–18.2)
WBC	9520.0 (7470.0–11,800.0)	9800.0 (7050.0–11,760.0)
Respiratory rate	20 (16–24)	24 (20–32)
Heart rate	52 (44–64)	64 (49–86)
Temperature (°C)	37.6 (37.4–38.0)	37.6 (37.2–38.0)
Lactate (mmol/L)	2.0 (1.3–3.4)	2.4 (1.6–3.7)

**Table 4:** Results from a multivariable logistic regression model identifying factors associated with survival in 138 colic horses.

Variables	Category	*p* Value	OR	CI 95%	
Type of colic	Obstructive non-strangulating colic	<0.001	11.80	3.80	37.00
	Strangulating colic	Reference			
Age		<0.004	0.91	0.84	0.97

OR, odds ratio; CI 95%, confidence interval.

## Data Availability

The data presented in this study are available on request from the corresponding author.

## References

[1] Curtis L., Burford J.H., England C.G.W., Freeman S.L. (2019). Risk factors for acute abdominal pain (colic) in the adult horse: A scoping review of risk factors, and a systematic review of the effect of management-related changes. PLoS ONE.

[2] Mair T.S., Smith L.J. (2005). Survival and complication rates in 300 horses undergoing surgical treatment of colic. Part 2: Short-term complications. Equine Vet. J..

[3] Moore J.N., Vandenplas M.L. (2014). Is it the systemic inflammatory response syndrome or endotoxemia in horses with colic?. Vet. Clin. Equine Pract..

[4] Sheats M.K. (2019). A Comparative Review of Equine SIRS, Sepsis, and Neutrophils. Front. Vet. Sci..

[5] Roy M.F., Kwong G.P.S., Lambert J., Massie S., Lockhart S. (2017). Prognostic Value and Development of a Scoring System in Horses with Systemic Inflammatory Response Syndrome. J. Vet. Intern. Med..

[6] Latson K.M., Nieto J.E., Beldomenico P.M., Snyder J.R. (2005). Evaluation of peritoneal fluid lactate as a marker of intestinal ischaemia in equine colic. Equine Vet. J..

[7] Shearer T.R., Norby B., Carr E.A. (2018). Peritoneal Fluid Lactate Evaluation in Horses with Nonstrangulating Versus Strangulating Small Intestinal Disease. J. Equine Vet. Sci..

[8] De Cozar M., Sherlock C., Knowles E., Mair T. (2020). Serum amyloid A and plasma fibrinogen concentrations in horses following emergency exploratory celiotomy. Equine Vet. J..

[9] Pihl T.H., Scheepers E., Sanz M., Goddard A., Page P., Toft N., Kjelgaard-Hansen M., Andersen P.H., Jacobsen S. (2016). Acute-phase proteins as diagnostic markers in horses with colic. J. Vet. Emerg. Crit. Care.

[10] Nocera I., Bonelli F., Vitale V., Meucci V., Conte G., Jose-Cunilleras E., Gracia-Calvo L.A., Sgorbini M. (2021). Evaluation of plasmatic procalcitonin in healthy, and in systemic inflammatory response syndrome (sirs) negative or positive colic horses. Animals.

[11] Kilcoyne I., Nieto J.E., Dechant J.E. (2020). Diagnostic value of plasma and peritoneal fluid procalcitonin concentrations in horses with strangulating intestinal lesions. J. Am. Vet. Med. Ass..

[12] Nocera I., Bonelli F., Meucci V., Rinnovati R., Spadari A., Intorre L., Pretti C., Sgorbini M. (2020). Evaluation of protein carbonyl content in healthy and sick hospitalized horses. Front. Vet. Sci..

[13] Archer D.C., Proudman C.J. (2006). Epidemiological Clues to Preventing Colic. Vet. J..

[14] Reeves M.J., Gay H.N.M., Hilbert B.J., Morris R.S. (1989). Association of Age, Sex and Breed Factors in Acute Equine Colic: A Retrospective Study of 320 Cases Admitted to a Veterinary Teaching Hospital in the USA. Prev. Vet. Med..

[15] Christophersen M.T., Dupont N., Berg-Sørensen K.S., Konnerup C., Pihl T.H., Andersen P.H. (2014). Short-Term Survival and Mortality Rates in a Retrospective Study of Colic in 1588 Danish Horses. Acta Vet. Scand..

[16] Straticò P., Varasano V., Palozzo A., Guerri G., Celani G., Revelant O., Petrizzi L. (2022). Retrospective study on risk factors and short-term outcome of horses referred for colic from 2016 to 2022. Vet. Sci..

[17] Matthews L.B., Sanz M., Sellon D.C. (2023). Long-term outcome after colic surgery: Retrospective study of 106 horses in the USA (2014–2021). Front. Vet. Sci..

[18] Fikri F., Hendrawan D., Wicaksono A.P., Purnomo A., Khairani S., Chhetri S., Purnama M.T.E. (2023). Incidence, risk factors, and therapeutic management of equine colic in Lamongan, Indonesia. Vet. World.

[19] Iglesias-García M., Rodríguez Hurtado I., Ortiz-Díez G., De la Calle Del Barrio J., Fernández Pérez C., Gómez Lucas R. (2022). Predictive Models for Equine Emergency Exploratory Laparotomy in Spain: Pre-, Intra-, and Post-Operative-Mortality-Associated Factors. Animals.

[20] Spadari A., Gialletti R., Gandini M., Valle E., Cerullo A., Cavallini D., Giusto G. (2023). Short-term survival and postoperative complications rates in horses undergoing colic surgery: A multicentre study. Animals.

[21] Southwood L.L. (2023). Early identification of intestinal strangulation: Why it is important and how to make an early diagnosis. Vet. Clin. Equine Pract..

[22] Long A.E., Southwood L.L., Morris T.B., Brandly J.E., Stefanovski D. (2024). Use of multiple admission variables better predicts intestinal strangulation in horses with colic than peritoneal or the ratio of peritoneal: Blood l-lactate concentration. Equine Vet. J..

[23] Fereig R.M. (2023). A review on equine colic: Etiology, differential diagnosis, therapy, and prevention. Ger. J. Vet. Res..

[24] Tharwat M., Al-Sobayil F. (2025). Equine colic: A comprehensive overview of the sonographic evaluation, diagnostic criteria, and management of different categories. Open Vet. J..

[25] Gillen A., Hassel D., Gonzalez S., Savage V., Bauck A., Freeman D., Archer D.C. (2025). Risk factors for equine strangulating lipoma colic: An international, case–control study. Equine Vet. J..

[26] Radcliffe R.M., Liu S.Y., Cook V.L., Hurcombe S.D., Divers T.J. (2022). Interpreting abdominal fluid in colic horses: Understanding and applying peritoneal fluid evidence. J. Vet. Emerg. Crit. Care.

[27] Corrie S., Chapman K., Schofield I., Mair T.S. (2024). Preliminary study to evaluate the use of fast abdominal ultrasonography of horses with colic in first opinion ambulatory practice. Equine Vet. Ed..

[28] Martin E., Sarkan K., Viall A., Hostetter S., Epstein K. (2024). Clinicopathologic Parameters of Peritoneal Fluid as Predictors of Gastrointestinal Lesions, Complications, and Outcomes in Equine Colic Patients: A Retrospective Study. Animals.

[29] Mickevičienė I., Mikalauskienė D., Miknienė Z. (2024). The prognostic importance of physiological and biochemical parameters in horses afflicted with colic. Open Vet..

[30] French N.P., Smith J., Edwards G.B., Proudman C.J. (2002). Equine surgical colic: Risk factors for postoperative complications. Equine Vet. J..

[31] Kos V.K., Kramaric P., Brloznik M. (2022). Packed cell volume and heart rate to predict medical and surgical cases and their short-term survival in horses with gastrointestinal-induced colic. Can. Vet. J..

[32] Macleod B.M., Wilkins P.A., McCoy A.M., Bishop R.C. (2025). Integration of machine learning and viscoelastic testing to improve survival prediction in horses experiencing acute abdominal pain at a veterinary teaching hospital. Equine Vet. J..

[33] Farrell A., Kersh K., Liepman R., Dembek K.A. (2021). Development of a colic scoring system to predict outcome in horses. Front. Vet. Sci..

[34] Mair T.S., Smith L.J. (2005). Survival and complication rates in 300 horses undergoing surgical treatment of colic. Part 1: Short-term survival following a single laparotomy. Equine Vet. J..

